# Pentavalent Vaccine‐Induced Immune Thrombocytopenia: A Case Report From Nepal

**DOI:** 10.1002/ccr3.70064

**Published:** 2025-01-06

**Authors:** Ramesh Khadayat, Sailesh Shrestha, Tilak Gautam, Sagar Rana Magar, Prashant Bhatta

**Affiliations:** ^1^ Patan Hospital Patan Academy of Health Science Lalitpur Nepal

**Keywords:** Nepal, Pentavalent‐vaccine immune thrombocytopenia, Petechiae, vaccine

## Abstract

Immune thrombocytopenia (ITP) is an autoimmune disorder marked by a low platelet count, leading to symptoms ranging from mild to severe bleeding. It can be triggered by various factors, including idiopathic origins, medications, malignancies, infections, and other autoimmune conditions. Though rare, ITP can also occur postvaccination. We report a case of a 3‐month‐old male who developed petechial rashes and mucosal bleeding following Pentavalent vaccination. This highlights the need for clinicians to recognize ITP as a potential complication of the Pentavalent vaccine. Educating parents about this rare but serious side effect is crucial.

## 1 Introduction

ITP is a rare autoimmune disease characterized by both platelet destruction and impaired megakaryocyte and platelet production, leading to increased risk of bleeding [1, 2]. The diagnosis is typically made by excluding the known causes of thrombocytopenia [3]. Generally, most of the patients are asymptomatic at the time of diagnosis, others may have mild symptoms and very few have severe symptoms [4]. In severe cases (platelet counts of < 20,000/μL), patients may experience bleeding complications such as intracranial and intestinal bleeding, which may be fatal [4]. ITP can be triggered by medication, infections, cancers, and autoimmune diseases. It is called primary immune thrombocytopenia when other causes or disorders that may be associated with thrombocytopenia are not identified (5), and it is called secondary if ITP is induced by other disorders or treatments [2]. Secondary causes of ITP are related to infection (viral), solid tumors, autoimmune disease (SLE), lymphoproliferative disorders, and drugs [3]. ITP has also been described in children following vaccinations, although this is exceedingly rare [5, 6]. The probability of developing ITP after vaccination and the mechanism of vaccine‐associated ITP are unclear. Reports from surveillance systems also face substantial reporting bias from different vaccination schedules, inconsistent surveillance, unattended cases, and challenges in diagnosing ITP accurately [7].

Pentavalent vaccine is a component of the national immunization program of Nepal, given at 6, 10, and 14 weeks of life. This vaccine provides protection against five life‐threatening illnesses, such as Diphtheria, Pertussis, Tetanus, Hepatitis B, and Hemophilus influenzae B. Together, this combination is called Pentavalent [8].

In this case report, a rare presentation of vaccine‐induced thrombocytopenia has occurred, probably due to pentavalent (DPT‐Hib). It has occurred after the third dose of Pentavalent and manifested with purpuric rashes in a 3‐month‐old baby.

## 2 Case History and Examination

### 2.1 Medical History

A 3‐month‐old male was admitted to our center with a chief complaint of gradually progressive petechial rashes on the face, abdomen, and limbs 3 days after receiving the third dose of the pentavalent vaccine. The child had no history of bleeding from other sites and did not develop features of anaphylaxis after vaccination. There was no history of altered level of consciousness, abnormal body movements, or other significant systemic symptoms. The family history of bleeding disorders was also noncontributory.

### 2.2 Physical Examination

On examination, the child was active and alert, with vital signs: temperature, 98.2 °F; heart rate, 120 beats/min; respiratory rate, 32 breaths/min; oxygen saturation, 97% in room air. Multiple petechial rashes were appreciated on the face, trunk, back, upper and lower limbs. Moreover, active mucosal bleeding was also noted on oral examination. The rest of the physical and systemic examination findings were unremarkable.

## 3 Methods

### 3.1 Investigation and Treatment

The child was evaluated for severe thrombocytopenia, and complete blood count showed a platelet count of just 7.5 thousand/μl of blood (normal range: 150–400 thousand/μl). Peripheral blood smear also showed reduced platelets, a manual count of 7500/cu.mm (normal range 150,000–400,000/cu.mm) with normal morphology of RBCs and WBCs. Other hematological parameters obtained on admission are shown in Table 1.

**TABLE 1:** Summary of investigation findings in a 3‐month‐old boy treated for immune thrombocytopenia at Patan Hospital, Nepal, 2024.

Test	Results	Reference
White blood cell	8.69 × 10^3^/μL	4.0–11 × 10^3^/μL
Hemoglobin	11.2 g/dL	13–18 g/dL
Platelets	7.5 × 10^3^/μL	150–400 × 10^3^/μL
Hematocrit	33%	40%–50%
Mean cell volume	71.9 ft	76–101 ft
Mean cell hemoglobin	24.4 pg	27–32 pg
PT/INR	13.8/1.06	9–11.8 s/0.8–1.2

**TABLE 2:** Summary of hematological parameters comparison on second, third, and fifth day of admission.

Test	Second day	Third day	Fifth day	Reference
White blood cell	6.21× 10^3^/μL	7.33 × 10^3^/μL	9.99 × 10^3^/μL	4.0–11 × 10^3^/μL
Hemoglobin	9.6 g/dL	9.9 g/dL	10.3 g/dL	13–18 g/dL
Platelets	37.5 × 10^3^/μL	75 × 10^3^/μL	157 × 10^3^/μL	150–400 × 10^3^/μL
Hematocrit	30%	30%	31%	40%–50%
Mean cell volume	76 ft	75 ft	75 ft	76–101 ft
Mean cell hemoglobin	24 pg	24 pg	25 pg	27–32 pg
PT/INR	—	—	—	9–11.8 s/0.8–1.2

Platelet concentrate was transfused, and empirical antibiotics were started after a septic workup was done. Antibiotics were stopped once urine and blood cultures were sterile. A diagnosis of immune thrombocytopenia due to the Pentavalent vaccine is made based on clinical probability and in the absence of other possible causes.

## 4 Results and Follow‐Up

Mucosal bleeding was stopped following transfusion of platelets concentrate. The child was observed in the hospital for any serious outcome, however, his petechial rashes began to disappear gradually and investigation showed a gradual rise in platelets as shown in Table 2. Serum vitamin B12 was 924 (normal range 239–931 pg/mL), and serum folate was 20 ng (normal range 2.78–20 ng/mL). The baby was discharged from the hospital once he was stable and platelets reached 157 × 10^3^/μl on the fifth day of hospital admission. He was stable without any issues on follow‐up after 1 week.

## 5 Discussion

This case report tries to show severe thrombocytopenia in the temporal context of a Pentavalent vaccine in a 3‐month‐old child, suggesting ITP as a rare complication. Child was admitted to the hospital for 5 days and had generalized petechial rashes and oral mucosal bleeding.

Immune thrombocytopenia is an autoimmune condition characterized by thrombocytopenia most commonly triggered by viral infection, however, vaccines can also induce ITP in rare cases. Studies done in Canada, USA, and Italy have shown that various vaccines, such as MMR (measles–mumps–rubella), diphtheria–tetanus–acellular pertussis, BCG, polio, influenza, hepatitis B virus, varicella, and enterovirus, are associated with increase risk of ITP [9, 10, 11, 12]. MMR vaccine is currently the only vaccine that has a cause–effect relationship with the development of ITP [7]. However, although many case reports have been published on ITP triggered by vaccination in children, ITP following Pentavalent (DPT‐Hib) vaccination is extremely rare, and only a few case reports are available [7, 13, 14]. The goal of the treatment is to stop bleeding which is primarily based on severity of bleeding rather than platelet counts [15]. The ITP in children is acute, short‐lived, and more likely to achieve spontaneous remission [16]. The first‐line treatment of ITP so far considered are corticosteroid and IV immunoglobulin, anti‐D immunoglobulin [17], however, the majority of the children with ITP do not require therapy with spontaneous resolution of disease [15, 16]. Therefore, in our case, we also managed the case conservatively.

In this case, although we were unable to establish a causal relationship between the Pentavalent vaccine and thrombocytopenia, this suggests that thrombocytopenia can potentially develop following the Pentavalent vaccine in children. Therefore, ITP triggered by Pentavalent vaccine should be considered in children, which can be manifested as severe thrombocytopenia with bleeding problems requiring immediate intervention.

## 6 Conclusion

Although Pentavalent vaccine‐induced ITP is rare, parents should be informed about this potential complication. Children should undergo meticulous physical examinations to detect any petechial rashes, purpura, or mucosal bleeding for prompt intervention. In cases, where petechial rashes appear after vaccination, ITP should be considered, and a history of recent Pentavalent vaccination should be noted in children with thrombocytopenia.

## Author Contributions

Ramesh Khadayat is involved in conceptualization, resources, writing – original draft, and writing – review and editing. Sailesh Shrestha and Tilak Gautam were involved in conceptualization, investigation, and writing – review and editing. Sagar Rana Magar and Prashant Bhatta were involved in investigation, resources, and writing – review and editing. The manuscript is reviewed and approved by all the authors.

## Consent

Written informed consent was obtained from parents for the publication of this case report. A copy of the written consent is available for review by the Editor‐in‐Chief of this journal on request.

## Conflicts of Interest

The authors declare no conflicts of interest.

## Data Availability

Data openly available in a public repository that issues datasets with DOIs.
